# Comparison of *SOX2* and *POU5F1* Gene Expression in Leukapheresis-Derived CD34+ Cells before and during Cell Culture

**DOI:** 10.3390/ijms24044186

**Published:** 2023-02-20

**Authors:** Małgorzata Świstowska, Paulina Gil-Kulik, Marcin Czop, Katarzyna Wieczorek, Arkadiusz Macheta, Alicja Petniak, Maria Cioch, Marek Hus, Mariusz Szuta, Mansur Rahnama-Hezavah, Bartosz J. Płachno, Janusz Kocki

**Affiliations:** 1 Department of Clinical Genetics, Medical University in Lublin, 20-080 Lublin, Poland; 2 Chair and Department of Oral Surgery, Medical University in Lublin, 20-081 Lublin, Poland; 3 Chair and Department of Hematooncology and Bone Marrow Transplantation, Medical University in Lublin, 20-081 Lublin, Poland; 4 Chair of Oral Surgery, Jagiellonian University Medical College, 31-155 Cracow, Poland; 5 Department of Plant Cytology and Embryology, Institute of Botany, Faculty of Biology, Jagiellonian University in Cracow, 30-387 Cracow, Poland

**Keywords:** CD34+ PBSCs, *SOX2*, *POU5F1*, cell culture

## Abstract

Bone marrow is an abundant source of both hematopoietic as well as non-hematopoietic stem cells. Embryonic, fetal and stem cells located in tissues (adipose tissue, skin, myocardium and dental pulp) express core transcription factors, including the *SOX2*, *POU5F1* and *NANOG* gene responsible for regeneration, proliferation and differentiation into daughter cells. The aim of the study was to examine the expression of *SOX2* and *POU5F1* genes in CD34-positive peripheral blood stem cells (CD34+ PBSCs) and to analyze the influence of cell culture on the expression of *SOX2* and *POU5F1* genes. The study material consisted of bone marrow-derived stem cells isolated by using leukapheresis from 40 hematooncology patients. Cells obtained in this process were subject to cytometric analysis to determine the content of CD34+ cells. CD34-positive cell separation was conducted using MACS separation. Cell cultures were set, and RNA was isolated. Real-time PCR was conducted in order to evaluate the expression of *SOX2* and *POU5F1* genes and the obtained data were subject to statistical analysis. We identified the expression of *SOX2* and *POU5F1* genes in the examined cells and demonstrated a statistically significant (*p* < 0.05) change in their expression in cell cultures. Short-term cell cultures (<6 days) were associated with an increase in the expression of *SOX2* and *POU5F1* genes. Thus, short-term cultivation of transplanted stem cells could be used to induce pluripotency, leading to better therapeutic effects.

## 1. Introduction

Bone marrow is an abundant source of both hematopoietic as well as non-hematopoietic stem cells. Non-hematopoietic stem cells are a heterogeneous group that includes mesenchymal stem cells, marrow-isolated adult multilineage inducible (MIAMI) cells, very small embryonic-like stem cells (VSEL), progenitor endothelial cells and multipotent progenitor cells. VSEL cells have the characteristics of embryonic, pluripotent stem cells. Other non-hematopoietic stem cells with many similarities co-create a microenvironment—a niche of hematopoietic stem cells [1,2]. Under physiological conditions, the majority of hematopoietic stem cells are located in the bone marrow (1–3% of mononuclear cells), while only a small proportion can be found in peripheral blood (0.01–0.1% of mononuclear cells) [3]. It is estimated that about 90–95% of hematopoietic stem cells located in the bone marrow are latent, and only 5–10% undergo active division [4]. Division and differentiation of hematopoietic stem cells result in the formation of progenitor cells for lymphoid and myeloid cell lines. Lymphoid line progenitor cells give rise to T-lymphocytes, B-lymphocytes, as well as NK cells. Differentiation of myeloid progenitor cells leads to the formation of megakaryocytes, erythrocytes, granulocytes and macrophages [5]. Hematopoietic stem cells under the influence of specific signals, such as granulocyte growth factor (G-CSF) or endogenous factors (hypoxia or trauma), may leave the niche going into the bloodstream [6,7,8,9]. G-CSF affecting the cells of the myeloid line. The myeloid line cells release proteolytic enzymes, such as cathepsin G, elastase and metalloproteinase-9, that digest adhesion molecules, as well as impair the interaction between chemokines, their receptors and intercellularity, leading to the release of hematopoietic stem cells into the bloodstream [10].

*SOX2* and *POU5F1* are the transcription factors responsible for self-renewal, pluripotency and initiation of differentiation pathway in stem cells. Thus far, there have been reports about *SOX2* gene expression in embryonic stem cells [11], perinatal tissue stem cells [12,13,14,15,16], breast milk-derived stem cells [17,18], dental pulp [19], adipose tissue [20,21], skin and myocardium [21], mesenchymal bone marrow stem cells [20,22], as well as nervous tissue stem cells and progenitor cells [23,24,25,26,27]. *POU5F1* expression has been found in embryonic stem cells [28], adipose tissue [29], bone marrow [21,30], Wharton’s jelly-derived stem cells [14], dental pulp [31], mesenchymal stem cells isolated from peripheral blood [32] and liver and heart cells [33]. 

The goal of this study was to demonstrate the expression of *SOX2* and *POU5F1* genes in CD34+ PBSCs from the graft material and to evaluate changes in this gene expression in cultured cells.

## 2. Results

### 2.1. Cytometric Analysis

To determine the content of CD34+ cells in each sample, cytometric analysis was performed using the Navios (Beckaman Coulter) flow cytometer.

**Figure 1:**
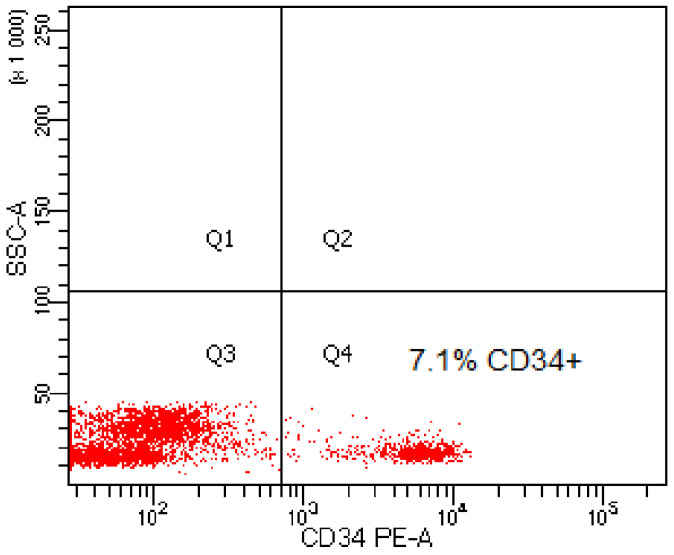
Exemplary cytogram presenting the content of CD34-positive cells in the graft material.

In the graft material obtained from 40 patients, the content of CD34+ cells was in the range of 6.1–13.35% (mean value 7.03% of CD34+ cells) (Table 1). Figure 1 shows an example of a cytogram showing the content of CD34+ cells in the graft material from one of the patients.

### 2.2. Analysis of Proliferation

**Figure 2:**
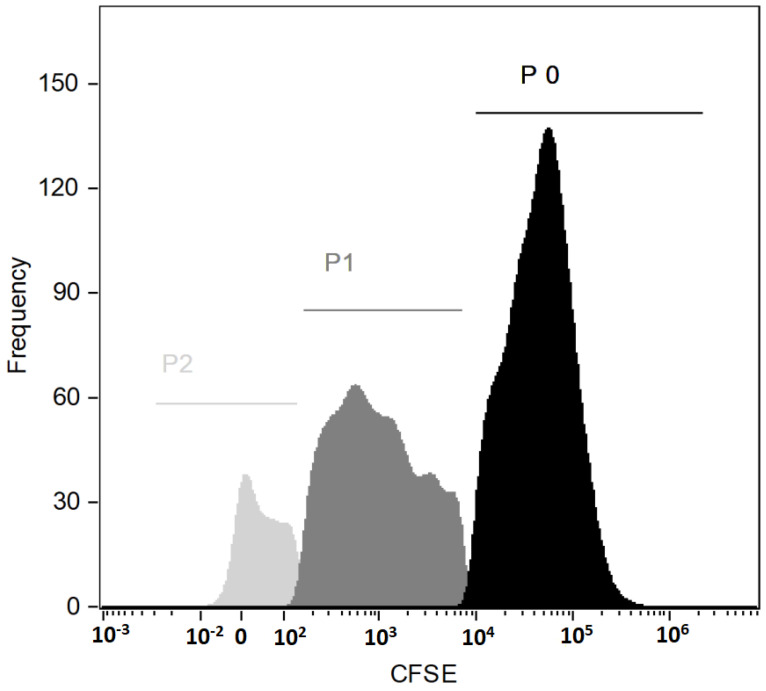
Histogram with three generations of PBSCs (emission peaks from CSFE dye bonded covalently to intracellular amines).

To determine cell cycle progression, analysis of the proliferation with a Cell Trace CSFE Kit was performed during cell culture. CFSE dye was added at the beginning of the cell culture and cytometric analysis was performed after 9 days of culture. Cytometric analysis showed the proliferating potential of the examined cells (Figure 2). The presented histogram shows three generations of cells: P0, P1 and P2, where P0 is the population of CD34 + cells obtained after magnetic sorting, while the populations P1 and P2 are the populations resulting from cell division during cell culture of the P0 population.

### 2.3. Analysis of Gene Expression (SOX2, POU5F1)

The one-way ANOVA test demonstrated statistically significant differences in the expression of the *SOX2* and *POU5F1* genes before and after cell cultures of CD34+ peripheral blood stem cells (*p* < 0.001). Testing showed statistically significant differences in the expression of the *SOX2* and *POU5F1* genes:

Before cell culture and after 3 days of culture (*p* = 0.0002);
Before cell culture and after 6 days of culture (*p* = 0.0002);
Before cell culture and after 9 days of culture (*p* = 0.0002).

**Figure 3:**
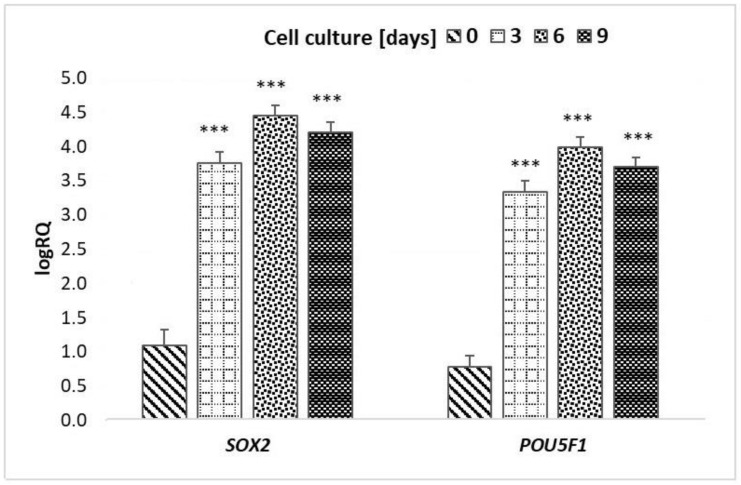
Mean and standard deviation values of LogRQ for the *SOX2* gene and *POU5F1* gene in peripheral blood stem cells prior to culture (day “0”) and on subsequent days of culture (3, 6 and 9 days). Explains: *** *p* < 0.001.

There were no statistically significant differences in the expression of the *SOX2* and *POU5F1* genes between particular days of culture (*p* > 0.05) (Table 2 and Figure 3).

The greatest differences in the expression of *SOX2* and *POU5F1* genes were observed between CD34+ PBSC before cell culture and CD34+ PBSC from the 6th day of culture (Figure 3).

## 3. Discussion

Before our studies, there was no available information about *SOX2* and *POU5F1* gene expression in CD34+ peripheral blood stem cells after mobilization with GCSF. In our studies, we were the first to demonstrate *SOX2* and *POU5F1* (also known as *OCT4*) gene expression in uncultured and cultured CD34+ peripheral blood stem cells after mobilization with GCSF.

In previous reports, researchers have shown changes in the expression of the *SOX2* and *POU5F1* genes during stem cell culture [13,14,15,19,20,34].

In an experiment by Yoon et al., mesenchymal stem cells isolated from Wharton’s umbilical cord jelly using the explant method exhibited *SOX2* and *OCT4* expression after 2 weeks of cell cultures, while in a study conducted by Gonzalez et al., expression of the *SOX2* and *OCT4* genes in human mesenchymal fetal stem cells from the second trimester persisted until 17th passage [12,35]. Cell cultures of adipose tissue stem cells carried out by Taha et al. showed a reduction in *SOX2* expression [34].

In our research, CD34+ peripheral blood stem cells showed a statistically significant increase in the expression of the *SOX2* and *POU5F1* genes until the 6th day of cell culture. However, there was a notable decrease in the expression of this gene after the 6th day of cell culture (*p* > 0.05). 

Similar results were obtained by Liu et al., who analyzed the expression of *SOX2* and *OCT4* in stem cells isolated from dental pulp. Cells from the second passage were characterized by greater expression of the *SOX2* and *OCT4* genes compared to cells after passage 0 or passage 7, suggesting a drop in expression of *SOX2* and *OCT4* in the course of cell culture and subsequent passages. An immunohistochemical examination of cells after passage 0 and passage 2 demonstrated the translocation of *SOX2* and *OCT4* from the cell nucleus into the cytoplasm, which might be associated with a gradual loss of pluripotency. Authors speculate that conditions under which cell cultures had been conducted might have influenced this loss of pluripotency [36].

In cell culture, the expression of the *SOX2* and *POU5F1* genes may also be influenced by other factors, such as culture density or the addition of factors stimulating cell division. Changes in the expression of the *SOX2* and *POU5F1* genes caused by cell culture density were described by Yoon et al. They examined the influence of cell culture density on the expression of core transcription factors—*SOX2*, *NANOG* and *OCT4* in stem cells isolated from the bone marrow. After 7 days of culture, the cells were passaged and divided into two separate cultures: “low density,” containing 17 cells/cm^3^ and “high density,” consisting of 5000 cells/cm^3^. Cell cultures were sustained for 12 days. Cells from the “low-density” culture were characterized by increased expression of the *SOX2*, *NANOG*, as well as *OCT4* genes [22]. Taha et al. analyzed changes in the expression of the *SOX2* and *OCT4* genes in stem cells isolated from murine adipose tissue induced by the addition of LIF (leukemia inhibitory factor) to cell cultures or transfection of stem cells with a vector containing miR-302. An increase in the expression of markers of pluripotency was observed in both cases. Possibly, the addition of LIF to cell culture led to the activation of JAK/STAT3, PI3/AKT and SHP2/MAPK signaling pathways responsible for the regulation of self-renewal and maintenance of pluripotency [34].

In opposition to these reports, in our studies, the changes in gene expression were not associated with the addition of any factors or changes in the density of cell cultures.

Appropriate modification of cell culture conditions (time and/or medium modifications) may contribute to an increase in *SOX2* and *OCT4* gene expression; thus, it might be associated with the induction of pluripotency in a cell without vector transfection [19].

## 4. Materials and Methods

### 4.1. Material

Transplant material containing PBSCs was obtained from 40 patients at the Department of Hematooncology and Bone Marrow Transplantation. Experiments were performed according to the protocol approved by the Bioethical Committee at the Medical University of Lublin (document no. KE-0254/334/2016).

### 4.2. Methods

#### 4.2.1. Peripheral Blood Stem Cells Isolation

PBSC isolation was conducted at the Department of Hematooncology and Bone Marrow Transplantation using the standard leukapheresis technique, according to the procedure described by Borowska et al., after the subcutaneous injection of G-CSF [37].

#### 4.2.2. Sample Preparation to Cytometric Analysis and Magnetic Separation

Samples containing Peripheral Blood Stem Cells were removed from liquid nitrogen and immediately placed in a water bath at 37 °C. After complete unfreezing, 20 mL of RPMI-1640 containing 10% of Fetal Bovine Serum was added (temperature 37 °C) and samples were centrifuged (10 min, 126× *g*). The supernatant containing the cryopreservative mixture was removed. Cells were suspended in a warmed-up PBS solution (Biomed, Lublin, Polland) and re-centrifuged (10 min, 126× *g*, temperature 37 °C). Following centrifugation, the supernatant was removed in order to get rid of the remaining cryopreservative agent. The remaining cells were suspended in 6 mL of warm PBS solution and subsequently divided into two portions. To remove cell clumps, the harvest apheresis was filtered through 30 µm nylon mesh. Samples intended for cytometric analysis and magnetic separation were centrifuged again. The supernatant was removed.

#### 4.2.3. Cytometric Analysis

Cells for cytometric analysis were suspended in 100 µL of PBS buffer containing no calcium or magnesium ions and transferred to cytometric probes. 10 μL of surface antibodies CD34- PE (Beckman Coulter, Brea, CA, USA) were added to each probe, and subsequently, samples were incubated at room temperature without access to light for 15 min. Afterward, 0.5 mL of PBS was added and cytometric analysis was performed on a Navios (Beckman Coulter) cytometer.

#### 4.2.4. Magnetic Cell Separation

The positive selection of CD34+ from leukapheresis product was conducted with the CD34 MicroBead Kit (Miltenyi Biotec, Bergisch Gladbach, Germany) on a MidiMACS Separator (Miltenyi Biotec, Gladbach, Germany) according to the manufacturer’s protocol using 300 µL of buffer, 100 µL of FcR Blocking Reagent and 100 µL of CD34 MicroBeads for up to 10⁸ total cells. After magnetic cell separation, the CD34-positive cells were divided into two equal portions.

#### 4.2.5. Cell Cultures

The samples for cell cultures were suspended in 15 mL of culture medium consisting of Fetal Bovine Serum (Gibco, Billings, MT, USA), Amphotericin B 250 μg/mL+ Penicillin/Streptomycin (100×) (PAA, Oberoesterreich, Austria) and RPMI-1640 with L-glutamine (Corning, New York, NY, USA) warmed up to 37 °C. PBSCs suspended in culture medium were transferred into culture vessels (TC Flask T25 for suspension culture (Sarstedt, Numbrecht, Germany)). Stem cells were cultured for 3, 6 and 9 days in a Brunswick New Galaxy 170R incubator (temperature 37 °C, 5% CO_2_ concentration, limited air supply). The culture medium was changed every 3 days.

#### 4.2.6. Cells Proliferation Analysis

Cells proliferation analysis was performed using Cell Trace CSFE Cell Proliferation Kit (Invitrogen, Carlsbad, CA, USA). During the setting of the cell cultures, a Cell Trace loading solution containing CFSE (carboxyfluorescein diacetate succinimidyl ester) was added according to the attached procedure for cells in suspension. The cytometric analysis of cell proliferation was done nine days later using a Flow Sight cytometer (Amnis, Seattle, WA, USA) and Amnis software.

#### 4.2.7. RNA Isolation

The portion intended for RNA isolation was centrifuged again. Stem cells were suspended in 500 μL of PBS and then centrifuged (10 min, room temperature, 126× *g*), and the supernatant was removed. A total of 500 μL of TRI-Reagent (Sigma, New York, NY, USA) was added to the cell pellet. Total RNA isolation was performed by the modified method of Chomczyński and Sacchi [38].

#### 4.2.8. Assessment of RNA Purity and Concentration

Probes containing isolated RNA under 75% ethanol were centrifuged (16,100× *g*, 15 min, 4 °C), and the supernatant (ethanol) was removed. The precipitant was dried at room temperature for 15 min. Subsequently, 12 µL of ultrapure water was added, pipetted and planted on ice.

RNA concentration in μg/μL was measured by applying 2 μL of the sample to a measurement pedestal of the Thermo Scientific Nanodrop 2000c Spectrophotometer. Pure, good-quality samples were selected for further analysis. The A260/280 coefficient was in the range of 1.8–2.

#### 4.2.9. Reverse Transcriptions

Reverse transcription of RNA was performed using a commercially available kit (High-Capacity cDNA Reverse Transcription Kit, Life Technologies, Carlsbad, CA, USA). 10 μL of a water-RNA solution containing 1 μg of RNA was used for the reaction. A 10 μL Master mix containing: 2 μL 10× RT Buffer, 0.8 μL 25 × DNTP Mix (100 mM), 2 μL 10× RT Random Primers, 1 μL RNase inhibitor, 1 μL MultiScribe Reverse Transcriptase and 3.2 µL of ultrapure water was added to each of the samples. The solution was mixed, briefly centrifuged and placed in the Veriti Dx Thermal Cycler (10 min 25 °C, 120 min 37 °C, 5 min 85 °C).

#### 4.2.10. *SOX2* and *POU5F1* Gene Expression

To determine the level of *SOX2* and *POU5F1* gene expression in peripheral blood stem cells, a Real-Time PCR reaction was performed (initial 10 min denaturation and 40 cycles: 15 s 95 °C, 60 s 60 °C) in StepOne Plus Real-Time PCR System. The PCR reaction mixture contains 1 µ cDNA, 1,25 µL gene-specific probe: *SOX2* (Hs0153049_s1, Applied Biosystems, Norwalk, CT, USA), *POU5F1* (Hs00999632_g1, Applied Biosystems, Norwalk, CT, USA) or *GAPDH* (Hs99999905_m1, Applied Biosystems, Norwalk, CT, USA) and Master Mix buffer (Applied Biosystems, Norwalk, CT, USA) *GAPDH* gene expression was used as an endogenous control. Based on the following formula, RQ = 2^−ΔΔCT^ [39], the relative gene expression (RQ) was calculated. For further analysis, log RQ was taken. Data analysis was performed using StepOne software v. 2.2.2 and Expression Suite software v.1.0.3.165 by Applied Biosystems.

#### 4.2.11. Statistical Analysis

Statistical analysis was carried out with Statistica v. 12 software using ANOVA rank test and multiple comparisons tests. Three levels of significance were determined: *p* < 0.05, *p* < 0.01 and *p* < 0.001.

All of the statistical details of the experiments can be found in the results sections and in Figure 3.

## 5. Conclusions

In summary, the examined cells exhibit *SOX2* and *POU5F1* gene expression both before cell culture and on the 3rd, 6th and 9th days of cultivation, reflecting their progenitor nature. Moreover, short-term cell culture (<6 days) on proper medium results in elevated expression of *SOX2* and *POU5F1* genes. Short-term cultivation of transplanted stem cells could be used to induce pluripotency, leading to better therapeutic effects in the proliferative stage.

## Figures and Tables

**Table 1:** The percentage content of CD34+ cells in the graft material obtained from 40 patients.

Content of CD34-Positive Cells in the Leukapheresis Products (%)	
Minimum	6.1
Maximum	13.35
Mean	7.03
Median	7.32
SD	3.3

**Table 2:** One-way ANOVA *p*-value for compared CD34+ PBSC groups for the analyzed targets (log RQ *SOX2* and logRQ *POU5F1*).

Gene	Group	*p*-Value (One-Way ANOVA; Unequal N HSD for Post-Hoc Tests)			
		PBSC	Cell Culture 3 Days	Cell Culture 6 Days	Cell Culture 9 Days
***SOX2* (logRQ)**	PBSC		0.0002 *	0.000 2*	0.0002 *
	cell culture 3 days	0.0002 *		0.3354	0.3448
	cell culture 6 days	0.0002 *	0.3354		0.9990
	cell culture 9 days	0.0002 *	0.3448	0.9990	
***POU5F1* (logRQ)**	PBSC		0.0002 *	0.0002 *	0.0002 *
	cell culture 3 days	0.0002 *		0.3105	0.4467
	cell culture 6 days	0.0002 *	0.3105		0.9839
	cell culture 9 days	0.0002 *	0.4467	0.9839	

* statistically significant differences in genes expression.

## Data Availability

The data used to support the findings of this study are included in the article.
